# Serum IL-12p40: A novel biomarker for early prediction of minimal change disease relapse following glucocorticoids therapy

**DOI:** 10.3389/fmed.2022.922193

**Published:** 2022-11-24

**Authors:** Mengqiu Bai, Jian Zhang, Xinwan Su, Xi Yao, Heng Li, Jun Cheng, Jianhua Mao, Xiayu Li, Jianghua Chen, Weiqiang Lin

**Affiliations:** ^1^Kidney Disease Center, The First Affiliated Hospital, Zhejiang University School of Medicine, Hangzhou, China; ^2^Department of Nephrology, The Fourth Affiliated Hospital, International Institutes of Medicine, Zhejiang University School of Medicine, Jinhua, Zhejiang, China; ^3^Institute of Translational Medicine, Zhejiang University School of Medicine, Hangzhou, Zhejiang, China; ^4^Department of Nephrology, Children’s Hospital, Zhejiang University School of Medicine, Hangzhou, Zhejiang, China

**Keywords:** minimal change disease, IL-12p40, biomarker, cytokines, glucocorticoids therapy

## Abstract

**Background:**

Minimal change disease (MCD) has a high recurrence rate, but currently, no biomarker can predict its recurrence. To this end, this study aimed at identifying potential serum cytokines as valuable biomarkers for predicting the risk of MCD recurrence.

**Materials and methods:**

Raybiotech 440 cytokine antibody microarray was used to detect the serum samples of eight relapsed, eight non-relapsed MCD patients after glucocorticoid treatment, and eight healthy controls. The differentially expressed cytokines were confirmed by enzyme-linked immunosorbent assay (ELISA) with serum samples from 29 non-relapsed and 35 relapsed MCD patients. The study used the receiver operating characteristic (ROC) curve analysis to investigate the sensitivity and specificity of a serum biomarker for predicting the MCD relapse.

**Results:**

Serum IL-12p40 levels increased significantly in the relapsed group. The Area Under the ROC Curve (AUC) of IL-12p40 was 0.727 (95%CI: 0.597–0.856; *P* < 0.01). The RNA-sequencing analysis and qPCR assay performed on the IL-12 treated mouse podocytes and the control group showed increased expression of podocyte damage genes, such as connective tissue growth factor (CTGF), matrix metallopeptidase 9 (MMP9), secreted phosphoprotein 1 (SPP1), and cyclooxygenase-2 (COX-2) in the former group.

**Conclusion:**

IL-12p40 may serve as a new biomarker for predicting the risk of MCD recurrence after glucocorticoid treatment, and it may be involved in the pathogenesis and recurrence of MCD.

## Introduction

Minimal change disease (MCD), characterized by intensive proteinuria leading to edema and intravascular volume depletion (1), is the primary cause of idiopathic nephrotic syndrome in 10–15% of adults. Glucocorticoids are the first-line therapy for adults with MCD since 1950 and continue to be the first-line therapy drug to date. (2) Over 90% of adult patients with MCD achieve a complete remission with glucocorticoid treatment; however, the relapse rates in adult MCD are high, with case series data showed that 56–76% of patients experienced at least one relapse after steroid-induced remission. (3–5) Although relapses are considered a serious clinical problem of MCD, there is currently no biomarker predicting the recurrence risk in glucocorticoids-treated MCD patients.

The pathogenesis of MCD remains to be elucidated; there could be several causes, with immune disorders possibly playing an essential role. Most of the earlier research on the pathogenesis of MCD primarily focused on the role of the immune system. (6) Several hypotheses about the pathogenesis of MCD have been proposed: T-cell disorder mediated by a circulating factor that alters podocyte function resulting in massive proteinuria, increased levels of several cytokines, and a “two-hit” theory that proposed the regulatory T-cell (Treg) dysfunction and CD80 (B7-1) induction. (7, 8) Many research focused on vascular endothelial growth factor (VEGF), interleukin-13 (IL-13), interleukin-12 (IL-12), interleukin-8 (IL-8), and tumor necrosis factor-α, (TNF-α) to understand the role of cytokines in MCD diseases. (9–13) Moderate podocyte VEGF164 overexpression during organogenesis results in congenital nephrotic syndrome, whereas VEGF164 overexpression after birth induces a steroid-resistant minimal change like-disease in mice. (9) Overexpression of IL-13 also induces minimal-change-like nephropathy in rats. (10) A significantly increased spontaneous and lipopolysaccharide (LPS)-stimulated release of IL-12 was detected in peripheral blood monocyte (PBM) cultures of MCD patients with the nephrotic syndrome (NS) compared with the normal controls; IL-12 levels increased during the active phase and normalized as the patients went into remission. (11) Early studies also suggested the involvement of IL-8 and TNF-α in the pathogenesis of MCD (12, 13). However, there is no systematic research conducted to identify effective cytokines that could predict the early recurrence of MCD.

In this study, a series of experiments was performed to explore the predictive role of serum cytokines in MCD relapse. A series of differentially expressed cytokines were identified by cytokine array. IL-12p40, verified *via* ELISA, distinguished 35 relapsed MCD patients from 29 non-relapsed MCD patients. The ROC curve analysis also demonstrated that the serum IL-12p40 was the best predictor of MCD relapse. Additionally, IL-12 treatment *in vitro* could cause changes in gene expressions related to podocyte injury.

## Materials and methods

### Patients

The study enrolled 64 participants between January 2013 and January 2016 from the Kidney Disease Center of the First Affiliated Hospital of Zhejiang University School of Medicine, China. All enrolled patients were diagnosed with primary MCD and treated with glucocorticoids. After their discharge from the hospital, the patients were followed up regularly as out-patient, every 3 months for the first year and every 3–6 months for the rest of the follow-up period. We divided study subjects into two groups: non-relapsed and relapsed groups. Relapse was defined as increased protein excretion of > 3 g/day with generalized edema. The sample included patients with the following criteria: (1) first time diagnosed with primary MCD and no previous treatment with steroids and immunosuppressants, (2) > 15 years old, (3) patients treated with glucocorticoids and steroid-sensitive. The exclusion criteria were: (1) patients with proven or suspected secondary MCD on renal biopsy, (2) patients with systemic diseases (diabetes mellitus), infections (HIV, HBV, HCV, URI), or drug abuse, and (3) patients who developed steroid-resistance or steroid-dependence. The control group included eight healthy patients with no evidence or history of infection, systemic, or renal disease. Relapse was defined as increased protein excretion of > 3.5 g/day in patients who underwent a partial or complete remission.

### Serum sample collection

The blood samples were collected on the day of initial renal biopsy before the drug treatment. The whole blood sample (not anticoagulated) was placed at room temperature for half an hour, centrifuged at 2000 rpm for 10 min, followed by aliquoting the upper serum portion into a smaller volume. They were then stored in a refrigerator at –80^°^C.

### Cytokine screening using cytokine antibody array

Raybiotech 440 cytokine antibody array was carried out to screen differentially expressed cytokine in the serum. In brief, the microarrays were equilibrated to room temperature and dried at room temperature for 1–2 h. Microarrays were blocked with dilute buffer for 1 h at room temperature, then 85 μl of the sample were added and incubated on the proteome microarray at 4°C overnight. The microarrays were washed the next day, then the antibody mixture was added into the microarrays and incubated at room temperature for 2 h. The microarrays were rewashed and incubated with Cy3 streptavidin at room temperature for 1 h. The microarrays were scanned with InnoScan 300 Microarray Scanner (Innopsys). Data were analyzed by GSH-CAA-440 data analysis software.

### Measurement of serum cytokines

Serum levels of cytokines were assayed using commercially available ELISA kits (R&D Systems, Minneapolis, MN, USA) according to the manufacturer’s instructions. The respective standard curves were prepared from standard dilutions of each of the five ELISA kits of IL-12p40, pentraxin-3, C–C motif chemokine ligand 22 (CCL22), angiotensin I converting enzyme-2 (ACE-2), and receptor for advanced glycation endproducts (RAGE), and the sample concentration of each cytokine was determined. Finally, the OD450 was determined using a microplate reader (Molecular Devices M5, USA).

### Immunohistochemical staining and immunofluorescence staining

These procedures performed as previously described. (14, 15) The primary antibodies used were as follows: anti-IL12RB1 (13287-1-AP; Proteintech) and anti-Synapotodin (sc-515842; Santa Cruz).

### Mouse podocyte culture and treatment

The conditionally immortalized mouse podocyte cell line was donated by Professor Karlhans Endlich (University of Greifswald, Greifswald, Mecklenburg-Vorpommern, Germany). The cells were maintained in RPMI 1640 supplemented with 10% FBS, 100 U/ml penicillin, and 100 mg/ml streptomycin (Gibco-BRL, Gaithersburg, MD, USA). For proliferation, the podocytes were cultured in collagen type I-coated dishes in the presence of mouse recombinant IFN-γ (10 U/ml) at 33^°^C. For differentiation, the medium was switched to a non-IFN-γ medium for 10–14 days at 37^°^C. The podocytes were incubated with 400 ng/ml IL-12 (Peprotech Inc., Rocky Hill, NJ, USA) for 6 h for RNA-sequencing. The sequence results were verified by quantitative polymerase chain reaction (qPCR) with 400 ng/ml IL-12 treatment for 6 and 12 h.

### RNA deep sequencing and data analysis

RNA was extracted from the podocytes in the IL-12 treatment group and the control group. The total RNA was subjected to RNA-sequencing to determine mRNA expression patterns under the IL-12 treatment. Bioinformatics analyses, including raw data process, differential gene expression analysis, and Kyoto Encyclopedia of Genes and Genomes (KEGG) analysis, were conducted by Novogene (Beijing, China).

### RNA extraction and quantitative polymerase chain reaction analysis

The cells were collected and purified using ESscience RNA-Quick Purification Kit (YiShan Biotech, Shanghai, China). The RNA samples were reverse transcribed for mRNA level quantification *via* qPCR using HiScript III RT SuperMix and qPCR Master Mix (Vazyme). Supplementary Table 1 lists the primers for qPCR. Glyceraldehyde 3-phosphate dehydrogenase (GAPDH) was used as an internal control for CTGF, MMP9, SPP1, and COX-2 qPCR.

### Statistical analysis

SPSS 17.0 for Windows was used to perform statistical analyses, and all values were expressed as mean ± standard deviation (SD). Comparisons between groups were made using an unpaired *t*-test. *P*-values of < 0.05 were considered statistically significant.

## Results

### Screening of cytokines in the discovery cohort

**FIGURE 1:**
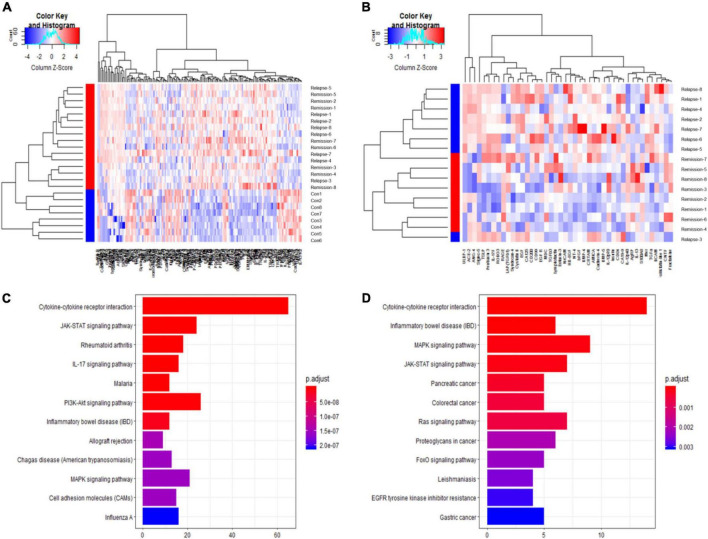
Raybiotech 440 cytokine antibody microarray results. The heat map analysis **(A,B)** revealed that cytokines could distinguish between minimal change disease (MCD) and healthy controls and had different expression patterns between non-relapsed MCD and relapsed MCD. The red and blue colors in the heat map depict higher and lower gene expressions, respectively. The color intensity indicates the magnitude of the expression differences. A fold change of 1.2 and an adjusted *P*-value of 0.05 were used as the screening criteria **(A)**. The differentially expressed proteins screened by *P*-adjust were too few, so the *P*-value was used as the screening criteria **(B)**. **(C)** Shows the Kyoto Encyclopedia of Genes and Genomes (KEGG) pathway analysis for differential expression proteins between MCD and healthy controls. **(D)** Shows the KEGG pathway analysis for differentially expressed proteins between non-relapse MCD and relapse MCD patients. The analysis showed the 12 most enriched pathways with the smallest adjusted *P*-value.

Serum cytokine microarray profiles of 16 patients in two groups [non-relapsed MCD (*N* = 8) and relapsed MCD patients (*N* = 8)] were collected using a single standard protocol and analyzed. The age distribution, gender, and other clinical characteristics are listed in Table 1. The serum uric acid in the relapsed MCD group was slightly higher than that in the non-relapsed MCD (*P* = 0.03). The other clinical parameters showed no significant difference between the two groups (*P* > 0.05). Eight healthy control also performed Raybiotech 440 cytokine antibody microarray, and the age, gender, and other clinical characteristics were matched. A total of 164 differentially expressed proteins were screened in the control group and the MCD group. Forty-four differentially expressed cytokines were found between the relapsed and non-relapsed groups (fold change > 1.2 and *P*-value < 0.05) (Figures 1A,B). We screened different expression proteins and classified them into the enrichment pathway using the KEGG pathway for further analysis. The results indicated an association of MCD with cytokine–cytokine receptor interaction, JAK-STAT, IL-17, PI3K-AKT, and MAPK pathways. The relapse of MCD was also associated with cytokine–cytokine receptor interaction, JAK-STAT, and MAPK pathways (Figures 1C,D). The protein chip results showed that the JAK-STAT and MAPK pathways play a crucial role in the development and relapse of MCD.

**TABLE 1:** Baseline characteristics of non-relapsed minimal change disease (MCD), relapsed MCD patients, and healthy controls with Raybiotech 440 cytokine antibody microarray.

Characteristics	Non-relapse MCD group (*N* = 8)	Relapse MCD group (*N* = 8)
Women, *n* (%)	75%	62.5%
Age at enrollment, yr	30 ± 7.25	24 ± 4.62
Proteinuria, g/d	5.93 ± 2.76	4.81 ± 3.7
Serum albumin, g/L	20.18 ± 4.16	20.96 ± 9.24
SCr, μmol/L	62.86 ± 12.3	72.63 ± 32.5
eGFR, mL/min per 1.73 m^2^	114.57 ± 15.32	119.74 ± 35.65
Fasting blood sugar mmol/L	4.35 ± 0.46	4.15 ± 0.59
Serum cholesterol, mmol/L	9.47 ± 2.54	9.16 ± 0.95
Serum triglyceride	2.11 ± 1.01	2.01 ± 0.86
Serum LDL, mmol/L	6.41 ± 2.5	5.14 ± 2.33
Serum uric acid, μmol/L	275.0 ± 61.27	368.13 ± 93.08

Data are presented as the mean ± SD.

### Validation of array results with enzyme-linked immunosorbent assay

**FIGURE 2:**
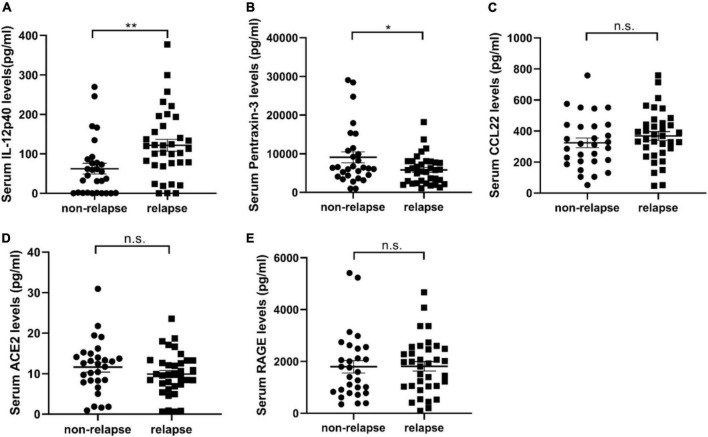
The validation results of serum cytokine antibody microarray by enzyme-linked immunosorbent assay (ELISA). **(A–E)** Show the expression of IL-12p40, pentraxin-3, CCL22, ACE, and RAGE in the serum samples of 35 relapsed and 29 non-relapsed minimal change disease (MCD) patients. The expression of IL-12p40 in the relapsed group was 121.7 ± 14.71 pg/ml, and the expression in the non-relapsed group was 62.46 ± 13.52 pg/ml (*P* = 0.005). The expression of pentraxin-3 in the relapsed group was 5817 ± 639.2 pg/ml, and the expression in the non-relapsed group was 9080 ± 1406 pg/ml (*P* = 0.029). The expression of CCL22 (*P* = 0.2965), ACE (*P* = 0.2527), and RAGE (*P* = 0.9565) in the serum samples of relapsed and non-relapsed groups showed no significant differences. *P < 0.05; **P < 0.01; n.s., not significant.

To validate the specific biomarkers for relapse of MCD, serum samples from 29 non-relapsed and 35 relapsed MCD patients (including the samples of eight non-relapsed and eight relapsed MCD patients used in serum cytokine microarray) were measured *via* ELISA. Table 2 lists the clinical information of the participants. Table 3 presents the differentially expressed cytokines between the non-relapsed and relapsed groups. Five cytokines (IL-12p40, pentraxin-3, CCL22, ACE-2, and RAGE) were chosen for ELISA validation based on cytokine antibody microarray results and literature review; these five cytokines could be related to the MCD recurrence mechanism. The levels of IL-12p40 and pentraxin-3 obtained from the ELISA results were consistent with the array results, confirming expression differences between the non-relapsed and relapsed MCD groups (*P* < 0.05), but the expression of CCL22, ACE-2, and RAGE had no significant differences between the non-relapsed and relapsed MCD groups (Figure 2).

**TABLE 2:** Baseline characteristics of non-relapse and relapse minimal change disease (MCD) patients for the array results validation.

Characteristic	Non-relapse MCD group (*N* = 29)	Relapse MCD group (*N* = 35)
Women, (%)	44.83%	37.14%
Age at enrollment, yr	35 ± 15	33 ± 18
Proteinuria, g/d	4.95 ± 2.76	4.18 ± 2.59
Serum albumin, g/L	19.76 ± 6.06	18.9 ± 5.78
SCr, μmol/L	89.89 ± 55.31	99.74 ± 73.30
eGFR, ml/min per 1.73 m^2^	99.19 ± 30.73	98.99 ± 41.08
Fasting blood sugar mmol/L	4.51 ± 0.79	4.48 ± 1.0
Serum cholesterol, mmol/L	8.77 ± 2.51	9.3 ± 2.76
Serum triglyceride	1.92 ± 0.76	2.06 ± 0.97
Serum LDL, mmol/L	5.96 ± 2.33	5.99 ± 2.2
Serum uric acid, μmol/L	341.38 ± 88.88	385.63 ± 108.57

Data are presented as the mean ± SD.

**TABLE 3:** The information of top 20 differentially expressed cytokines between non-relapse group and relapse group in Raybiotech 440 cytokine antibody microarray.

Protein ID	AveExp. Relapse	AveExp. Remission	*P*-value	Fold change
ULBP-1	2.8	7.8	0.0154	0.03
ACE-2	3.1	7.7	0.0080	0.04
Siglec-5	14.2	17.4	0.0490	0.1
ANG-4	4.3	7.4	0.0462	0.1
TSLP	11.9	14.1	0.0396	0.2
IL-17F	12.8	14.9	0.0304	0.2
Cadherin-4	10.6	12.6	0.0092	0.3
AMICA	10.4	12.4	0.0075	0.3
Pentraxin-3	12.3	13.9	0.0175	0.3
CRTAM	10.7	12.3	0.0374	0.3
IL-12p70	11.2	10.8	0.0346	1.3
IL-13	11.6	11.2	0.0255	1.3
AgRP	12.2	11.8	0.0298	1.3
S100A8	11.8	11.3	0.0145	1.4
Lymphotactin	8.8	8.2	0.0414	1.5
Eotaxin-3	9.2	8.6	0.0408	1.5
LAP	16.0	15.3	0.0115	1.6
CCL22	10.0	9.3	0.0379	1.7
RAGE	15.6	14.5	0.0003	2.1
IL-12p40	12.9	11.5	0.0098	2.6

### Serum IL-12p40 as an indicator of relapse of minimal change disease

**FIGURE 3:**
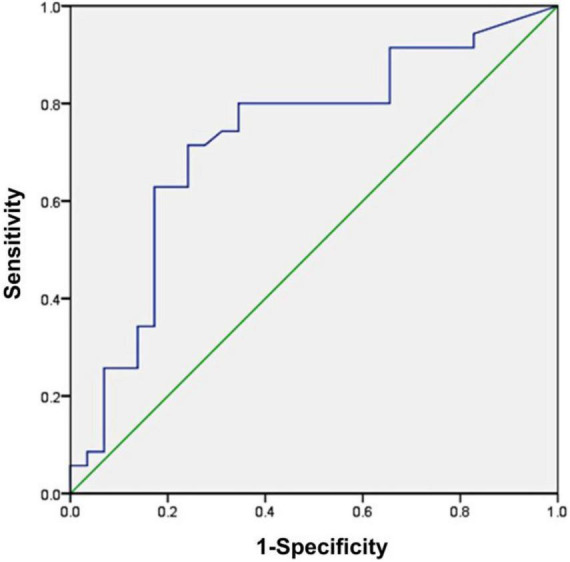
Receiver operating characteristic (ROC) curve analysis of serum IL-12p40 as a biomarker for the prediction of minimal change disease (MCD) relapse. The area under the ROC curve was 0.727 (95%CI: 0.597–0.856; *P* < 0.01), demonstrating that IL-12p40 was a suitable marker for predicting the relapse of MCD.

A conventional ROC curve measured the sensitivity and specificity of serum IL-12p40 that distinguished between the non-relapsed and relapsed MCD patients (Figure 3). The area under the ROC curve indicated the predictive value of this model. The results showed that the AUC of IL-12p40 was 0.727 (95%CI: 0.597–0.856; *P* < 0.01). The optimal cutoff level for IL-12p40 was 77.68 pg/mL, with a sensitivity and specificity of 71.4 and 75.9%, positive predictive value (PPV) of 78.1%, and negative predictive value (NPV) of 68.0% (*P* < 0.001). However, the expression differences of pentraxin-3 between the non-relapsed and relapsed MCD groups did not make sense.

### Interleukin-12 treatment promoted genes in the ECM–receptor interaction, PI3K-Akt, and focal adhesion signaling pathway

**FIGURE 4:**
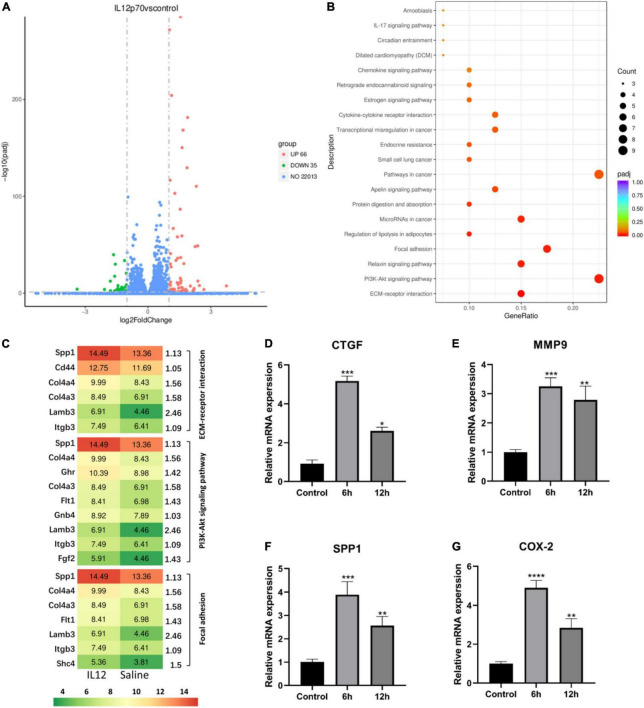
Differentially expressed genes in IL-12 treated mouse podocytes. **(A)** A volcano plot of differentially expressed genes in the IL-12-treated group versus the control group of podocytes. A fold-change > 2 and adjusted *P* < 0.05 were used as the screening criteria. **(B)** The Kyoto Encyclopedia of Genes and Genomes (KEGG) pathway enrichment analysis results of differentially expressed genes in the IL-12 treatment group and control group. The color depth is related to the adjusted *P*-value. **(C)** The related genes associated with ECM–receptor interaction, PI3K-Akt, and focal adhesion signaling pathways. **(D–G)** Quantification of relative mRNA expressions of the *CTGF*, *MMP9*, *SPP1*, and *COX-2* by qPCR. The podocytes in the IL-12-treated group were incubated with 400 ng/mL IL-12 for 6 h. **P < 0.01, ***P < 0.001, ****P < 0.0001.

Podocyte injury is a critical feature in MCD, so we investigated the deleterious effects of IL-12 on the podocytes. To detect the existence of IL12 receptors, we first performed qPCR assays of *IL12RB1* and *IL12RB2* (IL12 receptor genes) in human and mouse podocytes. The results showed that *IL12RB*1 and *IL12RB2* had some expression in these two cell lines (Supplementary Tables 2, 3). In addition, immunohistochemical (IHC) staining of a renal biopsy sample from the MCD patient showed positive staining of IL12RB1 on podocytes (Supplementary Figure 1). We also conducted immunofluorescence (IF) staining of a frozen section biopsy from an MCD patient using synaptopodin and IL12RB1 antibodies. IF results also demonstrated that IL12RB1 localized on podocytes (Supplementary Figure 2). Furthermore, the RNA-sequencing analysis was performed on mouse podocytes treated with IL-12 and saline, respectively. The results showed that compared with the control group, there were 101 differential genes in the IL-12 treatment group, of which 66 were upregulated and 35 were downregulated (Figure 4A). The KEGG pathway analysis indicated that the upregulated genes were top enriched in the ECM–receptor interaction, PI3K-Akt, and focal adhesion signaling pathways (Figure 4B). Figure 4C shows the genes associated with these three pathways. We verified the dramatically changed expression of genes, *CTGF*, *MMP9*, *SPP1*, and *COX2*, related to podocyte injury. The expression of four genes increased 3–5-folds in the podocyte cells treated with IL-12 for 6 h, compared to the control group; however, the increasing trend slightly decreased in the podocyte cells treated with IL-12 for 12 h (Figures 4D–G).

## Discussion

This study observed that the highly expressed IL-12p40 in the relapsed MCD patients could be used for early prediction of relapse of MCD-patients after the glucocorticoid treatment. IL-12p40, the common cytokine chain of IL-12 and IL-23, is vital in producing and potentially maintaining the Th1 responses and is reported to be involved in crescentic glomerulonephritis. (16–18) To our knowledge, no studies have explored the role of IL-12p40 in MCD to date. However, earlier research reported increased IL-12 levels in MCD patients during the active phase and its return to normal levels as the patients went into remission. (11) A study in Buffalo/Mna rats model found monocyte infiltration to be associated with an increase of TNFα, IL-1, and IL-12 transcripts before nephrotic syndrome recurrence. (19) It has been reported that the correlation between IL-12 and other glomerular diseases. The systemic lupus erythematosus (SLE)-like symptoms were exacerbated in lupus model mice treated with exogenous IL-12 and which were ameliorated in lupus model mice treated with an anti-IL-12 antibody (20). The clinical trial also supported ustekinumab, an IL-12/23 inhibitor, which functioned as a novel treatment in SLE (21). There are also some studies on the relationship between crescentic glomerulonephritis and IL-12. IL-12-/- mice were completely protected against experimental crescentic glomerulonephritis (22). IL-12p40 knockout mice failed to make a nephritogenic Th1 response and developed markedly reduced renal leukocytic infiltration and crescent formation (16). Our finding and these researches have supported that IL-12p40 is critical to pathologies associated with glomerular diseases.

This study further evaluated the role of IL-12 in MCD relapse *in vitro* by examining the expression of IL-12 in podocytes whose dysfunction is crucial in MCD pathogenesis. The KEGG pathway analysis performed on RNA-sequencing indicated enrichment of the upregulated genes in the ECM–receptor interaction, PI3K-Akt, and focal adhesion signaling pathways; the activation of these pathways was closely related to podocyte injury. Renal fibrosis is caused by excessive production and deposition of ECM, which is one of the main reasons for renal function loss. (23) The PI3K-Akt signaling pathway is one of the most important signal transduction pathways involved in regulating cell growth, proliferation, and differentiation. Furthermore, downregulated phosphorylation levels of PI3K-Akt could attenuate podocyte apoptosis. (24) It was noted that focal adhesion kinase (FAK) expression was upregulated in the LPS-induced rat podocyte injury models and that FAK activation was closely related to podocyte injury (25).

The RNA-sequencing and verification results showed that CTGF, SPP1, COX-2, and MMP9 were the most significantly upregulated genes in the IL-12 treatment group. It was proposed that the continuous expression of CTGF in podocytes is a key factor in promoting the progressive accumulation of glomerular ECM and glomerulosclerosis. (26) Additionally, CTGF is primarily involved in renal fibrosis and plays an important role in cell migration, adhesion, and inflammation. (27) Besides, SPP1 was influential in developing albuminuria in glomerular diseases; renal SPP1 mRNA increased in the LPS-induced albuminuria model and PAN-induced nephrotic rats. (28, 29) Overexpression of COX-2 could also cause podocyte injury. (30–33) Recent data indicated that MMPs played a role in many acute and chronic kidney pathophysiology. (34) Cytokines such as transforming growth factor-beta 1 (TGF-β1) could stimulate the MMP-9 secretion in podocytes that disrupted the integrity of podocyte connections and induced proteinuria. (35, 36) The four upregulated genes, *CTGF*, *SPP1*, *COX-2*, and *MMP9*, activated by IL-12 may cause podocyte injury.

To summarize, our study identified the serum cytokine (IL-12p40) to predict the recurrence of MCD treated with glucocorticoids, and it may serve as a new biomarker for the MCD relapse. The study is the first report of IL-12p40 as a biomarker of MCD recurrence, and more related research is needed in the future. One limitation of our current study: only small samples were used for biomarker screening and further validation. In the future, our group will collect more clinical samples to confirm these findings. Also, our research lacks *in vivo* animal experiments due to IL12 composed of p40 and p35 subunits. A single injecting recombinant IL-12p40 may not produce proteinuria and glomerular histology. In the future study, we can perform Adriamycin + IL-12p40 using a rodent model to test whether IL-12p40 can accelerate pathological progress. In addition, this research is a follow-up study with a varying specimen-collection time; a large multicenter prospective cohort study needs to be conducted in the future.

## Data availability statement

All data generated are included in this article and Supplementary material. The sequencing data is deposited in the GEO database under the accession ID GSE175968.

## Ethics statement

This study was reviewed and approved by the Ethics Committees of First Affiliated Hospital, Zhejiang University School of Medicine (Hangzhou, China). Written informed consent to participate in this study was provided by the participants’ legal guardian/next of kin.

## Author contributions

WL, XL, and JiC initiated and designed the study. MB and JZ performed most of the experiments. XS analyzed the RNA sequencing data and performed statistical analysis. XY, HL, JuC, and XL helped with patient recruitment. MB and WL wrote the initial manuscript. XL, JiC, WL, and JM revised and commented on the manuscript. All authors contributed to the article and approved the submitted version.
