# Supplementary material for: Serum IL-12p40: A novel biomarker for early prediction of minimal change disease relapse following glucocorticoids therapy
Source: Front Med (Lausanne). 2022 Nov 24;9:922193. doi: 10.3389/fmed.2022.922193 (PMC9729255; doi:10.3389/fmed.2022.922193)
Supplement: Supplementary file 1 [file Data_Sheet_1.docx]

**Serum IL-12p40: a novel biomarker for early prediction of minimal change disease relapse following glucocorticoids therapy**

Mengqiu Bai^1,2,3#^, Jian Zhang^1#^, Xinwan Su^1,2^, Xi Yao^1^, Heng Li^1^, Jun Cheng^1^, Jianhua Mao^4^, Xiayu Li^1^*, Jianghua Chen^1^*, and Weiqiang Lin^1,2^*

^1^Kidney Disease Center, the First Affiliated Hospital, Zhejiang University School of Medicine, Hangzhou 310003, Zhejiang, China.

^2^International Institutes of Medicine, Department of nephrology, the Fourth Affiliated Hospital, Zhejiang University School of Medicine, Jinhua 322000, Zhejiang, China.

^3^Institute of Translational Medicine, Zhejiang University School of Medicine, Hangzhou 310029, Zhejiang, China.

^4^Department of Nephrology, Children's Hospital, Zhejiang University School of Medicine, Hangzhou 310003, Zhejiang, China.

# Mengqiu Bai and Jian Zhang contribute equally to this manuscript.

*Corresponding author:

Dr. Weiqiang Lin, Zhejiang University School of Medicine, Tel/Fax: 86-571-86971990, Email: wlin@zju.edu.cn; or Dr. Jianghua Chen, Zhejiang University School of Medicine, Tel: 86-571-87236996, Fax: 86-571-87236188, Email: chenjianghua@zju.edu.cn; or Dr. Xiayu Li, Zhejiang University School of Medicine, Tel: 86-571-87236996, Fax: 86-571-87236188, Email: xy1313@sina.com.

**Supplementary materials**

**
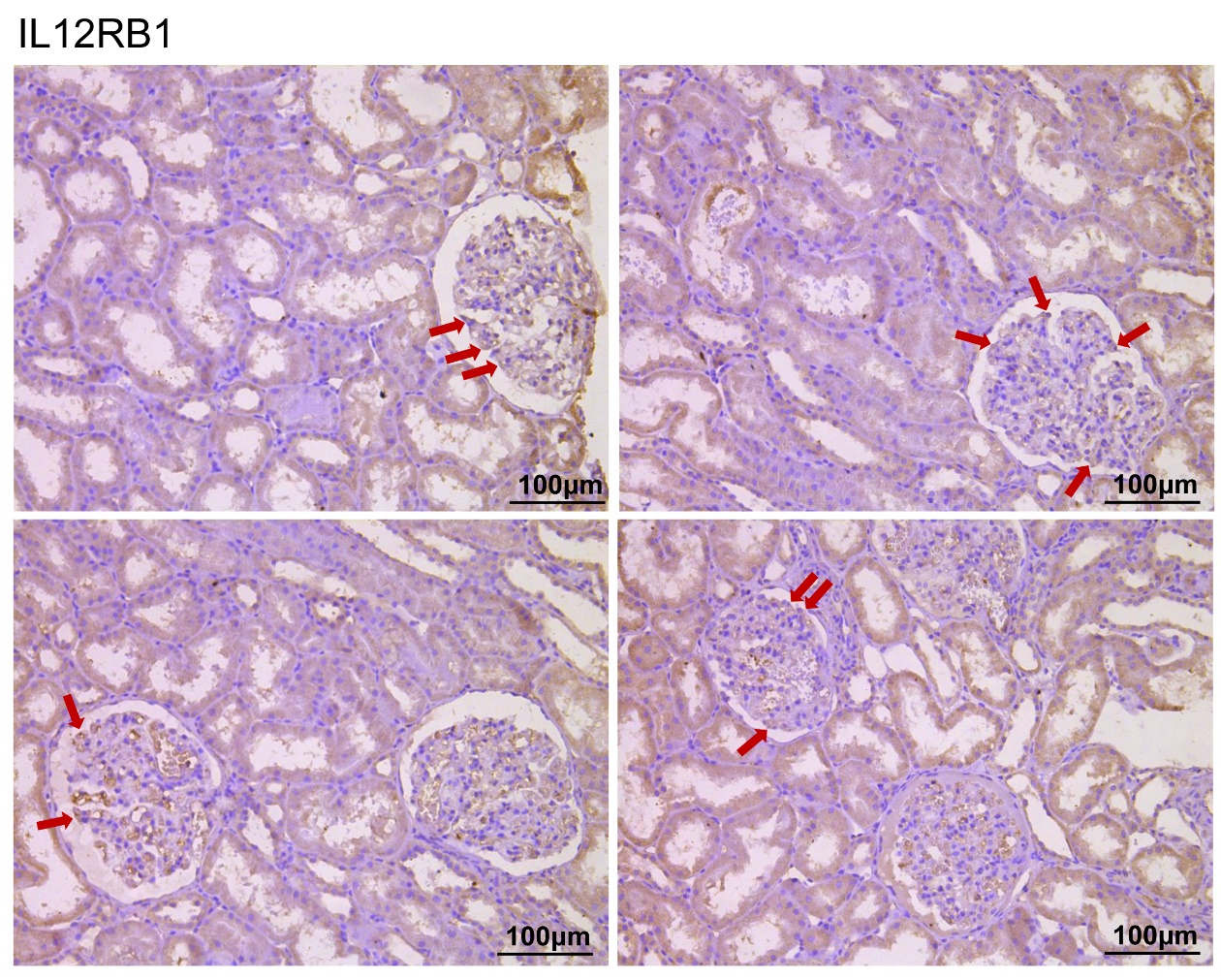
**

Figure S1

IL12RB1 localizes in glomerular podocyte. Representative images of IHC staining showed IL12RB1 staining (arrows) in podocytes from renal biopsy samples of the MCD patient.

**
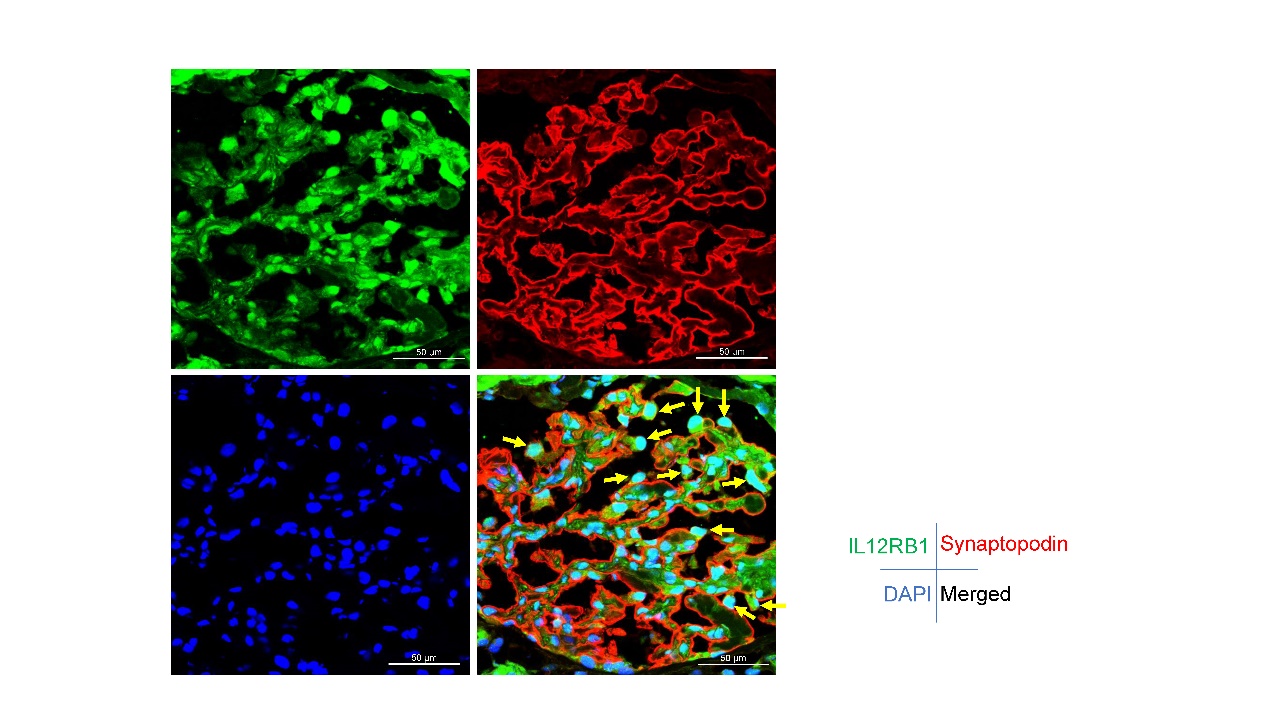
**

FIGURE S2

IL12RB1 localizes in glomerular podocyte. Representative images of IF staining showed IL12RB1 staining (Green) in podocytes from renal biopsy sample of the MCD patient. Green: IL12RB1; Red: synapotodin. Yellow arrow: a positive podocyte staining. Scale bar: 50 μm.

**TABLE S1** **Primers used for qPCR.**

| Primer Name | Sequence | |
| --- | --- | --- |
| m-GAPDH-F  m-GAPDH-R  m-CTGF-F | | TGGATTTGGACGCATTGGTC  TTTGCACTGGTACGTGTTGAT  GGGCCTCTTCTGCGATTTC |
| m-CTGF-F | | ATCCAGGCAAGTGCATTGGTA |
| m-SPP1-F  m-SPP1-R  m-COX-2-F  m-COX-2-R  m-MMP9-F  m-MMP9-R  m-IL12RB1-F  m-IL12RB1-R  m-IL12RB2-F  m-IL12RB2-R  h-IL12RB1-F  h-IL12RB1-R  h-IL12RB2-F  h-IL12RB2-R  h-ATCB-F  h-ATCB-R | | CTGGCAGCTCAGAGGAGAAG  TTCTGTGGCGCAAGGAGATT  CCAGCACTTCACCCATCAGTTT  TCTGTCCAGAGTTTCACCATAAATG  CTGGACAGCCAGACACTAAAG  CTCGCGGCAAGTCTTCAGAG  CGTGTCGGTGAGGAACCAAA  CCACTCTGACTCCCACGC  ACATGAGGCTGACCCAACTG  GAAGATGAGTGGGCGGAGAG  TAGGGACCTGAGATGCTATCG  CCCGGAGCTAAGGCAACAC  CCAGCCTCAGCTCTGTGAAA  GGGCTGCAGGCTGTTTATTG  AGCGAGCATCCCCCAAAGTT  GGGCACGAAGGCTCATCATT |

**TABLE S2 qPCR results of *ll12rb1* and *ll12rb2* in mouse podocytes**

|  | *Gapdh* | *Il12rb1* | *Il12rb2* |
| --- | --- | --- | --- |
| Ct | 18.27 | 30.35 | 30.26 |

**TABLE S3 qPCR results of *IL12RB1* and *IL12RB2* in human podocytes**

|  | *ACTB* | *IL12RB1* | *IL12RB2* |
| --- | --- | --- | --- |
| Ct | 13.35 | 29.72 | 33.93 |
